# From “invisible” to “audible”: Features extracted during simple speech tasks classify patient-reported fatigue in multiple sclerosis

**DOI:** 10.1177/13524585241303855

**Published:** 2024-12-17

**Authors:** Alyssa Nylander, Nikki Sisodia, Kyra Henderson, Jaeleene Wijangco, Kanishka Koshal, Shane Poole, Marcelo Dias, Nicklas Linz, Johannes Tröger, Alexandra König, Helen Hayward-Koennecke, Rosetta Pedotti, Ethan Brown, Cathra Halabi, Adam Staffaroni, Riley Bove

**Affiliations:** UCSF Weill Institute for Neurosciences, San Francisco, CA, USA; UCSF Weill Institute for Neurosciences, San Francisco, CA, USA; UCSF Weill Institute for Neurosciences, San Francisco, CA, USA; UCSF Weill Institute for Neurosciences, San Francisco, CA, USA; UCSF Weill Institute for Neurosciences, San Francisco, CA, USA; UCSF Weill Institute for Neurosciences, San Francisco, CA, USA; ki elements GmbH, Saarbrücken, Germany; ki elements GmbH, Saarbrücken, Germany; ki elements GmbH, Saarbrücken, Germany; ki elements GmbH, Saarbrücken, Germany; F. Hoffmann-La Roche, Basel, Switzerland; F. Hoffmann-La Roche, Basel, Switzerland; UCSF Weill Institute for Neurosciences, San Francisco, CA, USA; UCSF Weill Institute for Neurosciences, San Francisco, CA, USA; UCSF Weill Institute for Neurosciences, San Francisco, CA, USA; UCSF Weill Institute for Neurosciences, San Francisco, CA, USA

**Keywords:** Fatigue, multiple sclerosis, outcome measurement

## Abstract

**Background:**

Fatigue is a major “invisible” symptom in people with multiple sclerosis (PwMS), which may affect speech. Automated speech analysis is an objective, rapid tool to capture digital speech biomarkers linked to functional outcomes.

**Objective:**

To use automated speech analysis to assess multiple sclerosis (MS) fatigue metrics.

**Methods:**

Eighty-four PwMS completed scripted and spontaneous speech tasks; fatigue was assessed with Modified Fatigue Impact Scale (MFIS). Speech was processed using an automated speech analysis pipeline (ki elements: SIGMA speech processing library) to transcribe speech and extract features. Regression models assessed associations between speech features and fatigue and validated in a separate set of 30 participants.

**Results:**

Cohort characteristics were as follows: mean age 49.8 (standard deviation (*SD*) = 13.6), 71.4% female, 85% relapsing-onset, median Expanded Disability Status Scale (EDSS) 2.5 (range: 0–6.5), mean MFIS 27.6 (*SD* = 19.4), and 30% with MFIS > 38. MFIS moderately correlated with pitch (*R* = 0.32, *p* = 0.005), pause duration (*R* = 0.33, *p* = 0.007), and utterance duration (*R* = 0.31, *p* = 0.0111). A logistic model using speech features from multiple tasks accurately classified MFIS in training (area under the curve (AUC) = 0.95, *R*^2^ = 0.59, *p* < 0.001) and test sets (AUC = 0.93, *R*^2^ = 0.54, *p* = 0.0222). Adjusting for EDSS, processing speed, and depression in sensitivity analyses did not impact model accuracy.

**Conclusion:**

Fatigue may be assessed using simple, low-burden speech tasks that correlate with gold-standard subjective fatigue measures.

## Introduction

Multiple sclerosis (MS) is a chronic neuroinflammatory disease commonly accompanied by neurological fatigue. Fatigue is a major “invisible” symptom in people with multiple sclerosis (PwMS) that can precede diagnosis and worsen quality of life. Current gold-standard measures of fatigue typically require retrospective review and self-reporting of previous fatigue over the past 4 weeks.^1^ In addition, “state” assessments of fatigue describe an individual’s state in the moment, but these may require repeated measures over time to more comprehensively measure an individual’s tendency to experience fatigue.^2^ However, both of these types of assessment do not necessarily provide objective or ecologically meaningful data on fatigue and function.

PwMS have commonly reported changes in speech and voice characteristics, and some studies have suggested that changes to voice and speech may be related to fatigue.^3–5^ Recent work using automated speech analysis in MS and other neurologic diseases^6–9^ has demonstrated the potential of this technology to serve as an objective, rapid, and unbiased tool, collecting digital speech biomarkers that could quantify functional outcomes which are important to patients, like fatigue.

The goal of this study was to evaluate whether acoustic and linguistic features, extracted using automated analysis, are associated with self-reported MS fatigue. We further evaluated the performance of these features across three brief speech tasks—both scripted and spontaneous. We hypothesized that automated speech analyses could identify features of speech that correlate with fatigue levels and could, therefore, act as digital biomarkers of fatigue. From the features available, features relating to jitter, pitch, formants, and pauses were hypothesized to be most sensitive to fatigue.

## Methods

### Participants

In an ongoing transdiagnostic digital phenotyping study, a convenience cohort of PwMS was recruited from the University of California, San Francisco (UCSF) Multiple Sclerosis and Neuroinflammation Center; data from the 84 individuals were analyzed as the training set and 30 individuals as the test set. Inclusion criteria were as follows: a diagnosis of MS by 2017 McDonald Criteria and Expanded Disability Status Scale (EDSS) ⩽ 6.5 because part of the broader digital phenotyping study involves a walking task on an automated gait analysis walkway. EDSS Functional System scores were not available. Exclusion criteria were as follows: inadequate visual, auditory, and motor capacity to operate tablet-based programs, other neurological or non-affective psychiatric disorders.

### Procedure

All study procedures were approved and in accordance with the ethical standards of the Committee for Human Research at the University of California at San Francisco (institutional review board (IRB) No. 21-33227). Written informed consent was obtained from all participants. Participants completed speech tasks, cognitive assessments, and patient-reported outcomes (PROs) within one visit. All study assessments were administered by trained study staff.

### Speech task

Speech was recorded during three tasks:

- Scripted reading task (Grandfather Passage—a public domain text that is frequently used to generate speech samples);
- Description task (Image Description—prompt: “You will see a picture. Please describe this picture in detail, as if you were talking to someone who does not see the picture”); and
- Personal narrative task (Morning Routine—prompt: “Please describe what you do in the morning to get ready for your day.”).

While some participants completed all three tasks, others (<10%) completed only one or two due to time constraints. The order of the tasks was always the same when multiple tasks were performed. The total duration of time in acquiring speech for these tasks was approximately 3 minutes.

### Automated speech analysis

Speech was processed using a proprietary automated speech analysis pipeline (ki elements: SIGMA speech processing library)^10–13^ to transcribe speech and extract both acoustic and linguistic features.

- Jitter relates to glottic pulses or vibrations of the glottal folds, with reduced control on vocal fold vibrations resulting in a higher percentage of jitter and a harsh, hoarse, and rough voice quality. Shimmer refers to the same perturbation but relates to the amplitude of the sound wave.
- Pitch, characterized as the spectral F0 peak, is the fundamental frequency at which vocal cords vibrate.
- Formants are resonances above F0 and represent local spectral maximums resulting from an acoustic resonance of the human vocal tract. Variability of the first three formants across time strongly correlates with the range of motion of the jaw and tongue and overall muscle tenseness. Formant bandwidths increase with the presence of a glottal chink, common in breathy voices.
- Pause-related features reflect the duration, standard deviation (*SD*), and frequency of pauses, or silent spaces between speech utterances (utterance duration is also measured).

### PROs

Participants completed the Modified Fatigue Impact Scale (MFIS), a validated and gold-standard measure of self-reported fatigue in PwMS.^1^ The total score of the MFIS ranges from 0 to 84, with physical, cognitive, psychosocial subscores. Prior studies have used a total score of 38 as a cutoff to discriminate fatigued from non-fatigued individuals;^14,15^ this performs well as a cutoff for fatigue in most cases, but may underestimate fatigue in people with higher education, men <24 years, or women 65–74.^16^ In addition, participants completed the Hospital Anxiety and Depression Scale—Depression (HADS-D) to assess mood, and Symbol Digit Modalities Test (SDMT) to assess processing speed as an aspect of cognition.

### Statistical analysis

Descriptive statistics and *t*-tests were performed to describe demographic characteristics of the cohort. Correlations between speech features and MFIS were assessed with univariate regression and reported as Pearson’s correlations. For feature selection, LASSO (least absolute shrinkage and selection operator) regression with leave-one-out cross-validation was implemented. The LASSO method was employed to regularize and reduce the feature set, minimizing multicollinearity and optimizing model sparsity. Leave-one-out cross-validation ensured that the selected features consistently improved model performance, enhancing generalizability and reducing overfitting. The most significant parameters were selected to generate a nominal logistic model for fatigue as a categorical measure. Models were created using speech features from individual speech tasks as well as combinations of speech tasks, and covariates were sequentially added to the model to assess model stability. Finally, the model was applied to stratified populations: non-depressed individuals (HADS-D < 8), individuals with normal (SDMT ⩾ 50) and low (SDMT < 50) processing speed, and individuals with mild to moderate disability (EDSS < 4) to assess generalizability.

## Results

### Demographics

From the entire cohort, 84 participants were included in the training analyses and 30 subsequently enrolled participants were designated as the test set. Characteristics of the training set were as follows: mean age 49.8 (*SD* = 13.6), 71.4% female, mean MS disease duration 9.9 years (*SD* = 7.4), 85.7% relapsing-onset MS, median EDSS 2.5 (range: 0–6.5), mean MFIS 27.6 (*SD* = 19.4), and 29.8% had MFIS > 38, that is, in “fatigue” range. Similar characteristics were seen in the test set. Table 1 summarizes their demographic and clinical characteristics.

**Table 1.** Clinical and demographic characteristics of the participants: a predominantly relapsing, low-disability group on disease-modifying therapy, representative of the clinic population, divided into a training and test/validation sets.

	Training (*n* = 84)	Validation (*n* = 30)
Sex—*n* (%)		
Female	60 (71.4)	23 (76.7)
Male	24 (28.6)	7 (23.3)
Age at examination—mean (*SD*)	49.8 (13.6)	52.3 (12.4)
Race/Ethnicity—*n* (%)		
Asian/Pacific Islander/Native American	2 (2.38)	6 (20)
Black	4 (4.8)	2 (6.7)
Hispanic or Latino	13 (15.8)	0 (0)
White Non-Hispanic	58 (69.1)	19 (63.3)
Other/Declined/Unknown	7 (8.3)	3 (10)
MS type—*n* (%)		
Relapsing	72 (85.7)	20 (66.7)
Progressive	12 (14.3)	8 (26.7)
Not specified	0 (0)	2 (6.7)
MS disease duration—mean (*SD*)	9.9 (7.4)	12.1 (8.3)
EDSS—median (range)	2.5 (0–6.5)	3 (0–6.5)
Disease-modifying therapy—*n* (%)		
Anti-CD20	51 (64.6)	23 (79.3)
First generation injectable	6 (7.6)	4 (13.8)
Other monoclonal antibody	6 (7.6)	1 (3.5)
Oral	16 (20.3)	1 (3.5)
MFIS—mean (*SD*)	27.6 (19.4)	27.5 (19.3)
MFIS—*n* (%) ⩾ 38	25 (29.8)	7 (23.3)
HADS-Depression—mean (*SD*)	3.7 (3.4)	3.4 (2.8)
HADS-Depression—*n* (%) ⩾ 8	12 (14.5)	2 (6.9)
SDMT—mean (*SD*)	48.2 (13.1)	45.0 (9.5)
SDMT—*n* (%) < 50	34 (54.9)	23 (79.3)

*SD*: standard deviation; MS: multiple sclerosis; MFIS: Modified Fatigue Impact Scale; SDMT: Symbol Digit Modalities Test; EDSS: Expanded Disability Status Scale.

### Associations with speech features: individual tasks

**Figure 1.**
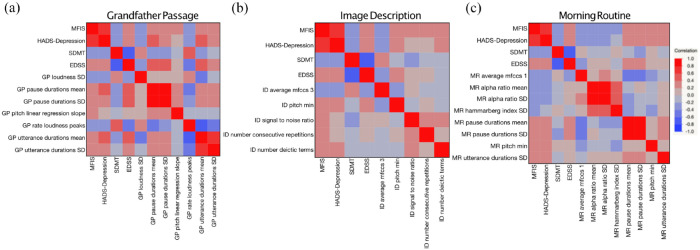
Speech features related to pauses, pitch, articulation, and loudness correlate with fatigue, as well as other patient-reported outcomes, in (a) Grandfather Passage, scripted reading speech task; (b) Image Description, description task; and (c) Morning Routine, personal routine task. Heat maps of Pearson’s correlations where red indicates positive correlation, and blue indicates negative correlation.

Correlations between MFIS and speech features were evaluated for each speech task (Table 2) and visualized as a heatmap (Figure 1). Notably, many of the features that correlated with MFIS also correlated with HADS-D and EDSS, and inversely correlated with SDMT. These covariates and their relationship to MFIS and speech features are discussed further below.

**Table 2.** MFIS showed modest correlations with individual speech variables associated with pitch, formants, pauses, and measures of articulation and loudness in the three individual speech tasks.

Grandfather Passage variable	*R*	*df* (*n* − 2)	*p*
GP loudness *SD*	−0.25	64	0.0394
GP pause duration mean	0.33	64	0.0072
GP pause duration *SD*	0.30	64	0.0160
GP pitch linear regression slope	0.26	64	0.0330
GP rate loudness peaks	−0.29	64	0.0186
GP utterance duration mean	0.30	64	0.0134
GP utterance duration *SD*	0.31	64	0.0111
Image Description variable	*R*	*df* (*n* − 2)	*p*
ID average MFCCS3	−0.29	71	0.0127
ID pitch min	0.32	71	0.0054
ID signal-to-noise ratio	0.26	71	0.0281
ID linguistic number of consecutive repetitions	0.28	71	0.0174
ID linguistic number of deictic terms	0.24	71	0.0396
Morning Routine variable	*R*	*df* (*n* − 2)	*p*
MR average MFCCS1	−0.25	71	0.0346
MR alpha ratio mean	−0.26	71	0.0260
MR alpha ratio *SD*	−0.31	71	0.0074
MR Hammarberg index *SD*	−0.23	71	0.0458
MR pause duration mean	0.30	71	0.0104
MR pause duration *SD*	0.32	71	0.0066
MR pitch min	0.26	71	0.0295
MR utterance duration *SD*	0.23	71	0.0494

Univariate regression was performed to assess Pearson’s correlations between speech task variables and Modified Fatigue Impact Scale (MFIS) total score. *SD*: standard deviation.

#### Pitch

Features related to pitch had moderate correlations with MFIS in all three speech tasks (Grandfather Passage: *R* = 0.26, *p* = 0.03; Image Description: *R* = 0.32, *p* = 0.005; and Morning Routine: *R* = 0.26, *p* = 0.03).

#### Pauses

Features related to pauses, including pause duration mean (Grandfather Passage: *R* = 0.33, *p* = 0.01; Morning Routine: *R* = 0.30, *p* = 0.01) and pause duration *SD* (Grandfather Passage: *R* = 0.30, *p* = 0.02; Morning Routine: *R* = 0.32, *p* = 0.01), were moderately correlated with MFIS. Utterance duration was moderately correlated with MFIS for the Grandfather Passage and Morning Routine tasks (mean—Grandfather Passage: *R* = 0.30, *p* = 0.01, *SD*—Grandfather Passage: *R* = 0.31, *p* = 0.01; *SD*—Morning Routine: *R* = 0.23, *p* = 0.05; signal to noise ratio—Image Description: *R* = 0.26, *p* = 0.03). Similar correlations were found between these speech metrics and the MFIS cognitive subscore.

#### Articulation and loudness

In addition, features related to articulation (MFCCS1, MFCCS3, Hammarberg index) and loudness (loudness *SD*, rate loudness peaks, alpha ratio mean and *SD*) were found to moderately correlate with MFIS.

### MFIS regression models

#### Single tasks

Next, we evaluated the ability of speech features to classify MFIS categorically, using a total score of 38 as a cutoff to discriminate fatigued from non-fatigued individuals.^14–17^ For feature selection, LASSO regression with leave-one-out cross-validation was implemented. Models using speech features from only one task explained moderate variance in MFIS score but could not be reproduced in the test set of participants, suggesting overfitting of the model (Supplemental Table 1).

#### Combined tasks

**Figure 2.**
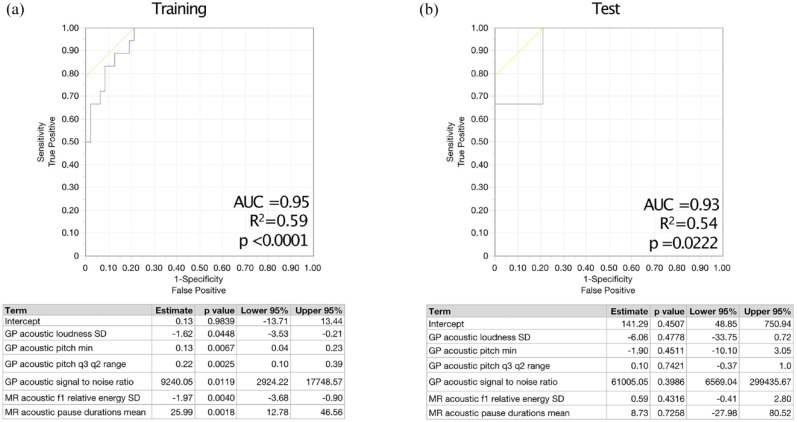
Models using speech features from more than one task robustly classify fatigued versus non-fatigued people with MS in both the (a) training and (b) test sets of participants.

Using speech features from more than one task improved model performance and separation in categorical models. Robust separation in fatigued versus non-fatigued categories was achieved using features from Grandfather Passage and Morning Routine (area under the curve (AUC) = 0.95, *R*^2^ = 0.59, *p* < 0.0001) (Figure 2(a)) and similarly robustly classified MFIS categories in the test set (AUC = 0.93, *R*^2^ = 0.54, *p* = 0.022) (Figure 2(b)).

Key covariates were sequentially added to the model to evaluate their effects on the model’s outcomes and stability, including HADS-D (depression), EDSS (disability), and SDMT (processing speed) (Table 3). HADS-D emerged as a significant covariate in explaining variance (estimate = −2.34, *p* = 0.015, 95% confidence interval (CI) = (−4.81, −0.78); SDMT and EDSS did not reach statistical significance as covariates. Then, a stratified analysis was performed limited to the non-depressed subsample (HADS-D < 8), and this did not change the accuracy of the model. In further exploratory analyses, the association between speech features and fatigue remained similar when restricting analyses to individuals with SDMT > 50 and separately EDSS < 4.

**Table 3.** Selected speech features from a scripted speech and spontaneous task robustly classify fatigued versus non-fatigued patients.

		AUC–ROC	*R* ^2^	Model *p*	DF/n	Misclassification rate	RASE	Estimate	Parameter *p*	95% CI
1	Model speech features—training set	0.95	0.59	<0.0001	6/65	0.1077	0.2814			
	Model speech features—test set	0.93	0.54	0.0222	6/25	0.1200	0.2967			
2	Model speech features	0.96	0.63	<0.0001	7/65	0.0923	0.2578			
	Age							0.07	0.1129	−0.01, 0.18
3	Model speech features	0.96	0.62	<0.0001	7/65	0.1077	0.2673			
	Gender							−1.36	0.1376	−3.37, 0.34
4	Model speech features	0.97	0.72	<0.0001	7/64	0.0781	0.2344			
	HADS-Depression ⩾ 8—No							−2.34	0.0153	−4.81, −0.78
5	Model speech features									
	*Stratified on: non-depressed (HADS-D* *<* *8)*	0.97	0.62	<0.0001	6/55	0.1273	0.2498			
6	Model speech features	0.95	0.61	<0.0001	7/64	0.1250	0.2713			
	EDSS ⩾ 4							1.70	0.1896	−0.77, 4.47
7	Model speech features									
	*Stratified on: Mild-moderate EDSS (EDSS* *<* *4)*	0.94	0.55	<0.0001	6/53	0.0943	0.2696			
8	Model speech features	0.95	0.60	<0.0001	7/49	0.1020	0.2725			
	SDMT							−0.04	0.5747	−2.61, 0.11
9	Model speech features									
	*Stratified on: normal processing speed (SDMT* ⩾ *50)*	0.99	0.81	<0.0001	6/40	0.05	0.1883			
	*Stratified on: low processing speed (SDMT* *<* *50)*	0.94	0.58	<0.0001	6/41	0.1463	0.3036			
10	Model speech features	0.99	0.74	<0.0001	10/63	0.0317	0.1966			
	Age							0.11	0.1269	−0.02, 0.28
	Gender							−2.04	0.2698	−7.03, 0.85
	HADS-Depression ⩾ 8							−2.31	0.0979	−5.84, −0.12
	EDSS ⩾ 4							2.087	0.2598	−1.20, 6.79

LASSO (least absolute shrinkage and selection operator) regression with leave-one-out cross-validation was implemented for feature selection, and most significant covariates were selected for model creation. Covariates were sequentially added to the model to assess model stability. The model was applied to stratified populations of non-depressed (HADS-D < 8), normal (SDMT ⩾ 50), and low (SDMT < 50) processing speed, and mild to moderate disability (EDSS < 4) participants to assess generalizability. CI: confidence interval; HADS-D: Hospital Anxiety and Depression Scale—Depression; SDMT: Symbol Digit Modalities Test; EDSS: Expanded Disability Status Scale; AUC: area under the curve; ROC: receiver operating characteristic; RASE: root average square error.

## Discussion

Fatigue is a commonly reported “invisible” MS symptom, that along with pain, depression, and cognitive impairment, greatly affects quality of life; furthermore, it is more predictive of self-reported health distress than visible symptoms like use of assistive devices.^18^ Speech, both in its acoustic and linguistic qualities, can provide subtle markers of well-being and can render fatigue more visible—or audible. The current analyses demonstrate that automated speech analysis of simple, low-burden, language tasks can approximate gold-standard subjective fatigue measures. Furthermore, they suggest that automatic speech analysis is viable as a tool to provide quantitative assessments of typically invisible or subjective patient experiences, which may facilitate better detection and subsequent targeted treatment.

Changes in both acoustic and linguistic qualities of speech have been evaluated in other neurodegenerative diseases. For example, in amyotrophic lateral sclerosis (ALS), speech is known to be slowed, with increased pauses and changes to voice quality, as well as having changes in language that may reflect cognitive changes.^19^ Similarly, higher rates of pausing and changes in speech prosody have been described in people with Parkinson’s disease (PD).^20^ In addition to previous gold-standard methods of speech analysis, which have typically involved speech-language pathologists manually assessing and scoring audio recordings, automated speech analysis of acoustic and lexical speech changes has more recently been applied to people with mild cognitive impairment (MCI), Alzheimer’s disease (AD), frontotemporal dementia (FTD), and primary progressive aphasia (PPA), particularly with regard to changes in cognitive abilities.^21–25^

Although changes to speech and language are common in MS, changes in these domains have overall not been appreciated as a major measure of dysfunction and have been less frequently studied, perhaps in part because presentations of disability can be so heterogenous in MS.^26,27^ Comparisons of speech between PwMS and healthy controls have yielded consistent differences across multiple studies. In particular, acoustic features relating to articulation, frequency, jitter, speech rate, and pauses have been consistently shown to differentiate between PwMS of varying degrees of severity and healthy controls.^6,28–30^ Notably, many of these speech features also appear to be relevant to fatigue. In the current analyses, speech features from multiple types of speech tasks were found to be indicative of fatigue—in particular, features relating to pauses, pitch and formants, and articulation. This intuitively makes sense based on these prior clinical observations. Similarly, another group, using the same automated speech pipeline on a German-speaking population of PwMS, found speech features related to pauses and pitch were predictive of fatigue as assessed by the Fatigue Scale for Motor and Cognitive Functions (FSMC) in a machine learning model using only a free speech task.^12^ This cross-linguistic demonstration of the importance of these features in assessing fatigue suggests there may be some underlying network or speech processing that is similar across language differences. This automated speech analysis pipeline may be scalable to other metrics and disease conditions as it has previously been validated in healthy controls and patients with PD, PSP, and ALS.^13^ This work demonstrates that automated speech analysis is capable of identifying the acoustic speech features clinically relevant to fatigue in PwMS. However, given that only 30% of participants had MFIS scores in the “fatigued” range, which is lower than has been generally previously reported, this may limit the generalizability of the results. As patients with progressive disease and high EDSS typically report higher fatigue levels, this more relapsing low-disability population may not be fully representative.

Depression and fatigue are often highly correlated in PwMS. Consequently, depression may confound the assessment of fatigue, complicating the interpretation of fatigue levels.^14,31^ Notably, while depression was found to be significant covariate in this model, the model retained predictive validity in a stratified non-depressed subset. This suggests that, while depression contributes significantly to the speech features related to fatigue, the model remains stable and generalizable to non-depressed populations, supporting the notion of central fatigue distinct from depression in PwMS.

There may also be differences in the number and types of words PwMS use with regard to personal conversational narratives, and objective naming impairment is known to be common in PwMS and tip-of-the-tongue phenomena.^32,33^ In addition, PwMS with cognitive impairment are noted to have changes in speech and articulation rate, as well as increased frequency and length of pauses.^30,34^ Three different speech tasks were used in this study: reading a scripted passage, describing a novel image, and generating a personal narrative—each likely utilizing different brain networks. These varied tasks likely challenged particular speech networks that are more impaired for PwMS. Interestingly, using features from two speech tasks improved the model performance, suggesting that querying speech by engaging multiple types of networks involved in language can increase sensitivity to detecting subtle changes related to fatigue. In this study, cognition as assessed by SDMT was not a significant covariate in the model. SDMT is a measure of processing speed that is generally accepted as a measure of cognitive performance in PwMS, but may not be sensitive to language-specific cognitive deficits—future work should investigate the interaction between these deficits and speech features in assessment of fatigue.

Changes in speech features have also been found to correlate with disability in other functional domains (particularly cerebellar dysfunction) and with quality of life measures in PwMS.^7,35,36^ While EDSS levels did not influence the associations identified in these analyses, including cerebellar and brainstem subscores would be more sensitive in excluding the effect of dysarthria from the assessment of fatigue.

In future work, speech tasks and automated speech analysis could potentially be used as an objective output in assessing fatigability, that is, objectively demonstrated deterioration of physical or cognitive function with activity, in addition to fatigue (a subjective feeling of lack of energy).^37^ For instance, some speech tasks generate a higher cognitive load, leading to more strain on an individual’s processing capacities, particularly for an already-fatigued patient. Thus, some speech tasks may be more sensitive to detecting fatigability. For example, dual-task performance has been shown to be sensitive to MS-related fatigue in some studies and is diminished in PwMS who have cognitive impairment.^38,39^ It has previously been suggested that speech tasks and the degree of the cognitive load have a greater effect on MS patients than on healthy controls.^40^ It is not known which speech tasks might prove most fatiguing or challenging to PwMS—prior work suggests that reading may have a reduced cognitive load compared with spontaneous speech, but it is not clear if this holds true in all PwMS, particularly those with cognitive impairment, and further research into this question is warranted.^30,41^

The current work substantially advances ongoing efforts to identify and promote objective measures of fatigue through three innovations. First, we used automatic speech detection and analysis tools that improve scalability and reproducibility. This is in contrast to traditional surveys, which typically require a trained administrator and scorer to improve the accuracy and reliability of responses, even if they can be self-administered. Second, the speech tasks, including spontaneous speech tasks (as opposed to only scripted reading tasks or repetitions of vowel sounds), increase the ecological validity by more closely resembling “real life” speech performance—and are brief and low-burden to patients, potentially increasing the likelihood of their participation and engagement. Finally, having an objective measure of subjective symptoms will help to reduce bias and improve reproducibility across research studies seeking to reduce fatigue. The era of precision medicine and telehealth medicine necessitates that we have sensitive tools to track individual outcomes important to patients: novel tools and metrics such as those afforded by automated speech analysis will increase opportunities to assess patient well-being simply, digitally, and remotely.

### Limitations

Limitations to this study include that it was performed at a single site, a quaternary referral center, albeit one with significant geographic, economic, and racial/ethnic diversity. Indeed, overall the population had relatively low disability and low fatigue and thus may not be generalizable to all PwMS. In addition, the order of the speech tasks was not counterbalanced, and therefore, it is possible that fatigability could have increased over the course of the tasks. While EDSS levels were analyzed in the model, brainstem/cerebellar subscores were not available to be specifically interrogated. Finally, this data set is cross-sectional, and further longitudinal work will be necessary to determine whether speech performance is stable over time, how to determine clinically meaningful differences in speech features, and demonstrate a more direct relationship between these features and changes in fatigue.

## Conclusion

Fatigue is a commonly reported “invisible” MS symptom that may be assessed using simple, low-burden, language tasks that correlate with gold-standard subjective fatigue measures. Automated speech analysis has real-world applicability to provide quantitative assessments of typically invisible or subjective patient-reported experiences.

## Supplemental Material

sj-docx-1-msj-10.1177_13524585241303855 – Supplemental material for From “invisible” to “audible”: Features extracted during simple speech tasks classify patient-reported fatigue in multiple sclerosis Supplemental material, sj-docx-1-msj-10.1177_13524585241303855 for From “invisible” to “audible”: Features extracted during simple speech tasks classify patient-reported fatigue in multiple sclerosis by Alyssa Nylander, Nikki Sisodia, Kyra Henderson, Jaeleene Wijangco, Kanishka Koshal, Shane Poole, Marcelo Dias, Nicklas Linz, Johannes Tröger, Alexandra König, Helen Hayward-Koennecke, Rosetta Pedotti, Ethan Brown, Cathra Halabi, Adam Staffaroni and Riley Bove in Multiple Sclerosis Journal
