# Supplementary material for: From “invisible” to “audible”: Features extracted during simple speech tasks classify patient-reported fatigue in multiple sclerosis
Source: Mult Scler. 2024 Dec 17;31(2):231–41. doi: 10.1177/13524585241303855 (PMC11789430; doi:10.1177/13524585241303855)
Supplement: sj-docx-1-msj-10.1177_13524585241303855 – Supplemental material for From “invisible” to “audible”: Features extracted during simple speech tasks classify patient-reported fatigue in multiple sclerosis [file sj-docx-1-msj-10.1177_13524585241303855.docx]

Supplementary Table 1. Models using features from only one speech task were able to classify fatigued vs non-fatigued participants explained moderate variance in MFIS, but could not be reproduced in the test set. GP = Grandfather Passage, ID = Image Description, MR = Morning Routine

|  | AUC-ROC | R^2^ | Df/n | P value |
| --- | --- | --- | --- | --- |
| GP – training set | 0.82 | 0.16 | 2/66 | 0.0018 |
| GP – test set | 0.52 | 0.01 | 2/25 | 0.8931 |
| ID – training set | 0.92 | 0.52 | 8/73 | <0.0001 |
| ID – test set | 0.84 | 0.27 | 8/25 | 0.4927 |
| MR – training set | 0.89 | 0.39 | 5/73 | <0.0001 |
| MR – test set | 0.68 | 0.09 | 5/26 | 0.7809 |
